# Development of a microfluidic photochemical flow reactor concept by rapid prototyping

**DOI:** 10.3389/fchem.2023.1244043

**Published:** 2023-08-07

**Authors:** Robin Dinter, Suzanne Willems, Thilo Nissalk, Oguz Hastürk, Andreas Brunschweiger, Norbert Kockmann

**Affiliations:** ^1^ Laboratory of Equipment Design, Department of Biochemical and Chemical Engineering, TU Dortmund University, Dortmund, Germany; ^2^ Medicinal Chemistry, Department of Chemistry and Chemical Biology, TU Dortmund University, Dortmund, Germany; ^3^ Department of Chemistry and Pharmacy, Institute for Pharmacy and Food Chemistry, Julius-Maximilians-University of Würzburg, Würzburg, Germany

**Keywords:** DNA-encoded chemistry (DEL), photochemistry, flow photoreactor concept, batch to flow, rapid prototyping, photoredox reaction

## Abstract

The transfer from batch to flow chemistry is often based on commercial microfluidic equipment, such as costly complete reactor systems, which cannot be easily tailored to specific requirements of technologies such as DNA-encoded library technology (DELT), in particular for increasingly important photochemical reactions. Customized photoreactor concepts using rapid prototyping technology offer a modular, flexible, and affordable design that allows for adaptation to various applications. In order to validate the prototype reactors, a photochemical pinacol coupling reaction at 368 nm was conducted to demonstrate the transfer from batch to flow chemistry. The conversion rates were optimized by adapting the design parameters of the microfluidic flow photoreactor module. Subsequently, the photoreactor module has been extended to an application with DNA-tagged substrates by switching to LEDs with a wavelength of 454 nm. The successful recovery of DNA confirmed the feasibility of the modular-designed flow photo reactor. This collaborative approach holds enormous potential to drive the development of DELT and flow equipment design.

## 1 Introduction

DNA-encoded library technology (DELT) provides a sustainable approach to small molecule screening. The technology is based on synthesizing libraries of a chimeric molecule built from a DNA strand that encodes covalently coupled small molecule. These small molecules are built through a combinatorial process of DNA-tagging and compound synthesis steps. Compound synthesis methods need to be high-yielding, robust, DNA- and water-compatible. Thus, the number of applicable reactions is limited, for instance, harsh reaction conditions need to be avoided (Satz et al., 2022). Recently, photochemically mediated reactions have been established on the DNA-barcode, giving access to a broadened chemical space in DELT (Kölmel et al., 2018; Kölmel et al., 2020; Kölmel et al., 2021; Patel et al., 2021).

Photochemistry has become increasingly important for synthesizing small molecules, as it enables chemical transformations under mild reaction conditions and with a broad functional group tolerance. It is based on specific excitation of a photo-active metal catalyst or dye with photons at a specific wavelength, initiating single-electron processes with molecules (Prier et al., 2013). A primary focus of this field is the relationship between the irradiation source and the interaction of the photons with the molecules during the reaction process (Noël, 2017; Rehm, 2020a; Svejstrup et al., 2021). In recent years, the transfer of batch chemistry to continuous flow has gained increasing interest. Continuous photoreactors significantly improve conversions and reaction times because they have a high surface-to-volume ratio that provides effective irradiation (Cambié et al., 2016; Noël, 2017; Rehm, 2020b; Sambiagio and Noël, 2020).

Combining photochemistry and continuous flow chemistry exhibits tremendous potential to distribute higher light rates throughout the reaction volume and increase conversion rates. Advances in 3D printing and the reductions in the cost of 3D-printer equipment and required hardware have made rapid prototyping of flow reactors accessible to many laboratories (Rossi et al., 2020). Rapid prototyping and modular-designed reactors enable the creation of numerous variations in a short construction time (Kockmann, 2016; Guba et al., 2019). The reactor can be flexibly adapted to other reaction conditions and wavelengths by replacing low-cost light emitting diode (LED) strips. The major advantage of rapid prototyping is the flexibility to develop tailored reactor concepts. Combined with open-source hardware, it is possible to build low-cost prototypes to investigate photochemistry challenges and the influencing conditions without relying on commercial and expensive photochemistry equipment (Guba et al., 2019; Rossi et al., 2020; González-Esguevillas et al., 2021).

Discontinuous photoreactors have been used in synthesizing DNA-encoded molecules (Kölmel et al., 2018; Kölmel et al., 2020), the use of continuous photoreactors for DNA-encoded reactions has not yet been reported in the literature. The pivotal aim of this work was to design a photochemical flow reactor for performing photochemical DEL reactions in flow, optimized by rapid equipment prototyping. First, batch and flow reactor prototypes were constructed using rapid prototyping, followed by validation conducting the photon-induced pinacol coupling as a model reaction. Next, the transfer of the batch photochemistry to a flow photoreactor module allowed the investigation of the relevant design parameters on the conversion of the model reaction. Due to the modular design, the number of LEDs, the coiling, and the inner diameter of the capillary reactor were varied. Finally, DNA recovery from a reaction setup was tested.

## 2 Experimental

### 2.1 Materials and instruments

Unless otherwise noted, chemicals were purchased from Sigma-Aldrich (Taufkirchen, Germany), Enamine (Kyiv, Ukraine), and Thermo Fisher Scientific (Karlsruhe, Germany). The modified DNA-strand was obtained from Ella Biotech (Fürstenfeldbruck, Germany). Conversion of the model reaction was determined by UV-Vis spectroscopy using an Agilent Cary 60 spectrophotometer (Agilent, United States). Prior to analysis, oligonucleotide-small molecule conjugates were precipitated by ethanol precipitation and analyzed by ion-pair reverse-phase high-pressure liquid chromatography (HPLC, Shimadzu Prominence) using a C18 stationary phase (Phenomenex, Gemini; 5 μm, C18, 110 Å, 100*4.6 mm) and a gradient of 10 mM aqueous triethylammonium acetate/methanol. In addition, oligonucleotides were analyzed by matrix-assisted laser desorption/ionization mass spectrometry (MALDI-MS, Bruker Daltonics) using a hydroxyl picolinic acid (HPA) matrix.

### 2.2 Experimental setup

For conducting the model reaction (Section 3.2), the protocol written by Volpe and Podlesny (Volpe and Podlesny, 2020) was used. The reactant solution was pumped using a 20 mL syringe (HSW Henke-Ject, Tuttlingen) fixed on the syringe pump VIT-FIT (HP) (Lambda, Czech Republic). A second syringe pump was used with a 2 mL syringe (HSW Henke-Ject, Tuttlingen) to flush the reactor by switching a three-port-valve (Bola, Germany) prior to it. The capillary reactor used fluorinated ethylene propylene (FEP) tubes with three different inner diameters, as described in Section 3.3, and was connected by IDEX fittings (IDEX, United States). The temperature measurement inside the reactors was measured using a Pt100A 10/10 sensor (Electronic Sensor, Heilbronn, Germany). The experimental setup for the DNA-tagged reactions was adapted from the literature-known procedures (Kölmel et al., 2021; Potowski et al., 2021) and performed under air.

## 3 Results and discussion

The construction of photoreactors used rapid prototyping methods (Section 3.1) since multiple parameters besides the reaction conditions (e.g., chemical concentration, temperature, or wavelength) were decisive for photoreactors. Additionally, to the reaction dependent setup, the design parameters, such as the geometry of the reactor and the interaction with the irradiation source significantly impact on the reaction conversion (Guba et al., 2019; Donnelly and Baumann, 2021; Svejstrup et al., 2021). The initial results from the prototypes were integrated into a flow photoreactor module (Section 3.3) to optimize the influencing design parameters that increased the model reaction conversions. The fully validated flow photoreactor was used with a modified LED wavelength in initial test reactions on DNA-tagged substrates to assess its applicability (Section 3.4).

### 3.1 Construction of prototypes for transferring batch to flow chemistry

**FIGURE 1:**
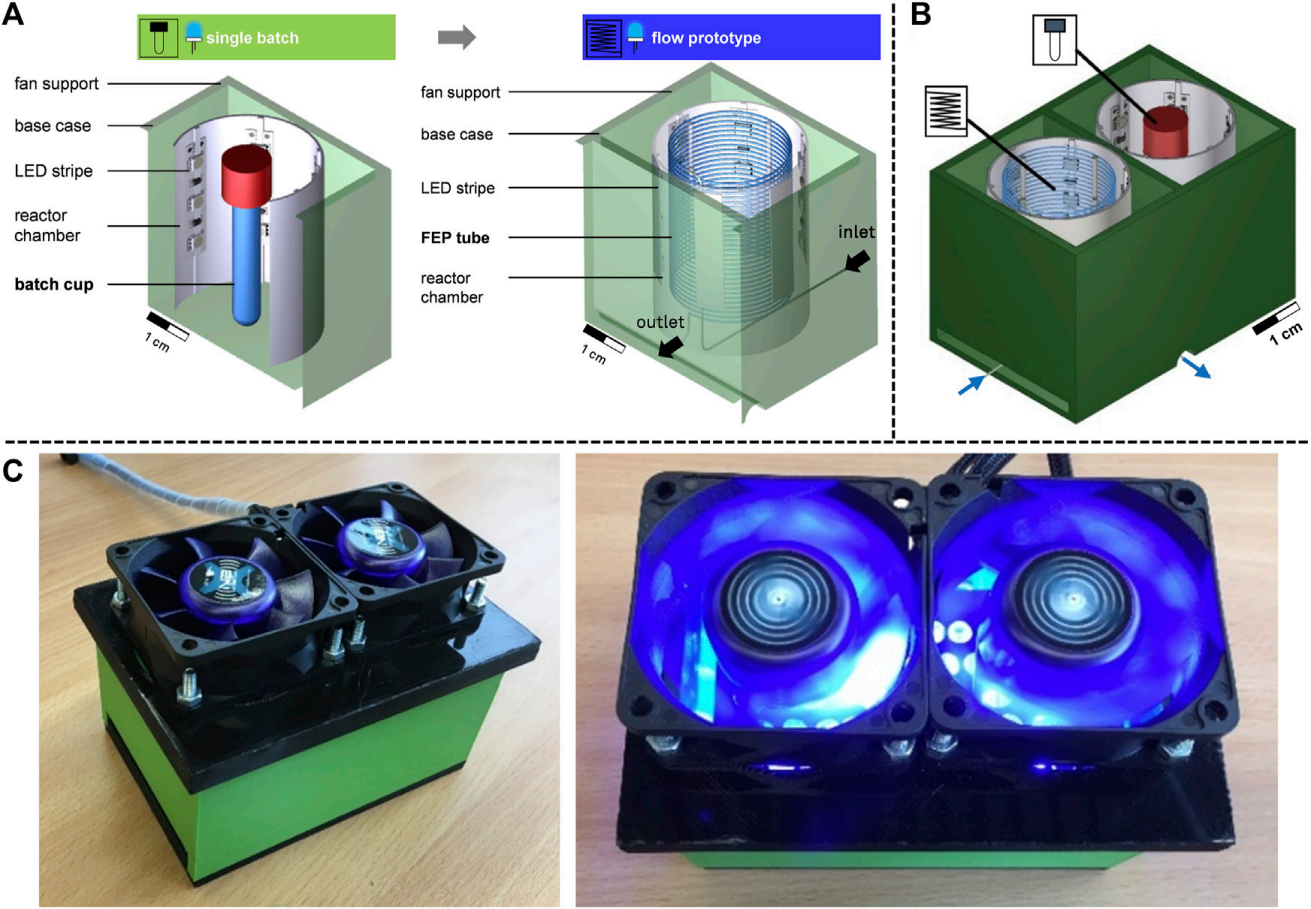
Prototype concepts. **(A)** Schematic overview of the single batch and the flow prototype. **(B)** Parallel conduction of model reaction to compare batch and flow photochemistry. **(C)** 3D-printed and constructed reaction chamber with fans for cooling.

Three prototype concepts (Figure 1) were initially designed using the computer-aided design (CAD) software Autodesk Fusion 360 (Autodesk, United States) to create technical drawings for fused deposition modeling (FDM) 3D printing with polylactide (PLA) on an Ultimaker S5 3D-printer (Ultimaker, Netherlands). The following requirements were placed on the prototypes. First, it was necessary to ensure that the reaction medium was uniformly irradiated to avoid over-irradiating the reactants and thus forming undesirable side-products. It was also essential to maintain a consistent energy input into the reactor and keep the distance between the irradiation source and the reaction medium as small as possible to facilitate targeted irradiation. Finally, appropriate cooling was essential to manage the temperature rise in the reactor caused by the electrical energy input from the irradiation source.

In the first concept (Figure 1), a batch prototype with a stirred batch microliter tube was developed that serves as a reference for the transfer to flow chemistry. LED strips are becoming increasingly popular for photochemistry due to their energy efficiency, space-saving design, low-cost and adjustable wavelength (Rehm, 2020a; Sambiagio and Noël, 2020). LED strips have a smaller volume and are more convenient to install than other light sources, such as incandescent lamps or neon tubes (Rehm, 2020a; Sambiagio and Noël, 2020). In this work, LED strips (BuyLEDStrip, Netherlands) with 18 individual LEDs were attached to the inside of the reactor chamber. The LEDs were powered by a 12 V, 3 A (36 W) power supply (Voltcraft, Germany) and the wavelength of the LEDs was 368 nm, which was required for the subsequent model reaction (Section 3.2). The material used for the base case was PLA (BASF, Inofil3D, Netherlands), which is UV-resistant, in contrast to acrylonitrile butadiene styrene (ABS), as another common material for 3D printing (Marek and Verney, 2016; Amendola et al., 2021). However, PLA has the disadvantage that the glass transition temperature is 57 °C (Marek and Verney, 2016), and the irradiation source leads to a temperature rise in the reactor. This was counteracted with convection air cooling by fans (Akasa, AK-FN076, China) on the base case, which provided the necessary cooling inside the reactor chamber. The discontinuous reactor chamber contains a batch microliter tube with a reaction volume of 2 mL. The photochemical flow prototype was developed based on a 4 m long coiled FEP tube with an inner diameter of 0.8 mm and an outer diameter of 1.6 mm (Figure 1). Thus, the flow photoreactor provided a comparable reaction volume (2.07 mL) to the batch reference with 2 mL. A direct comparison between batch and flow photochemistry was possible (Figure 1B), and the rapid prototyping approach allowed many variations to be made quickly to demonstrate the production of low-cost reactors using commercially available materials and open-source hardware. The prototypes were validated using the following model reaction in the next section.

### 3.2 Model reaction for validation

**FIGURE 2:**
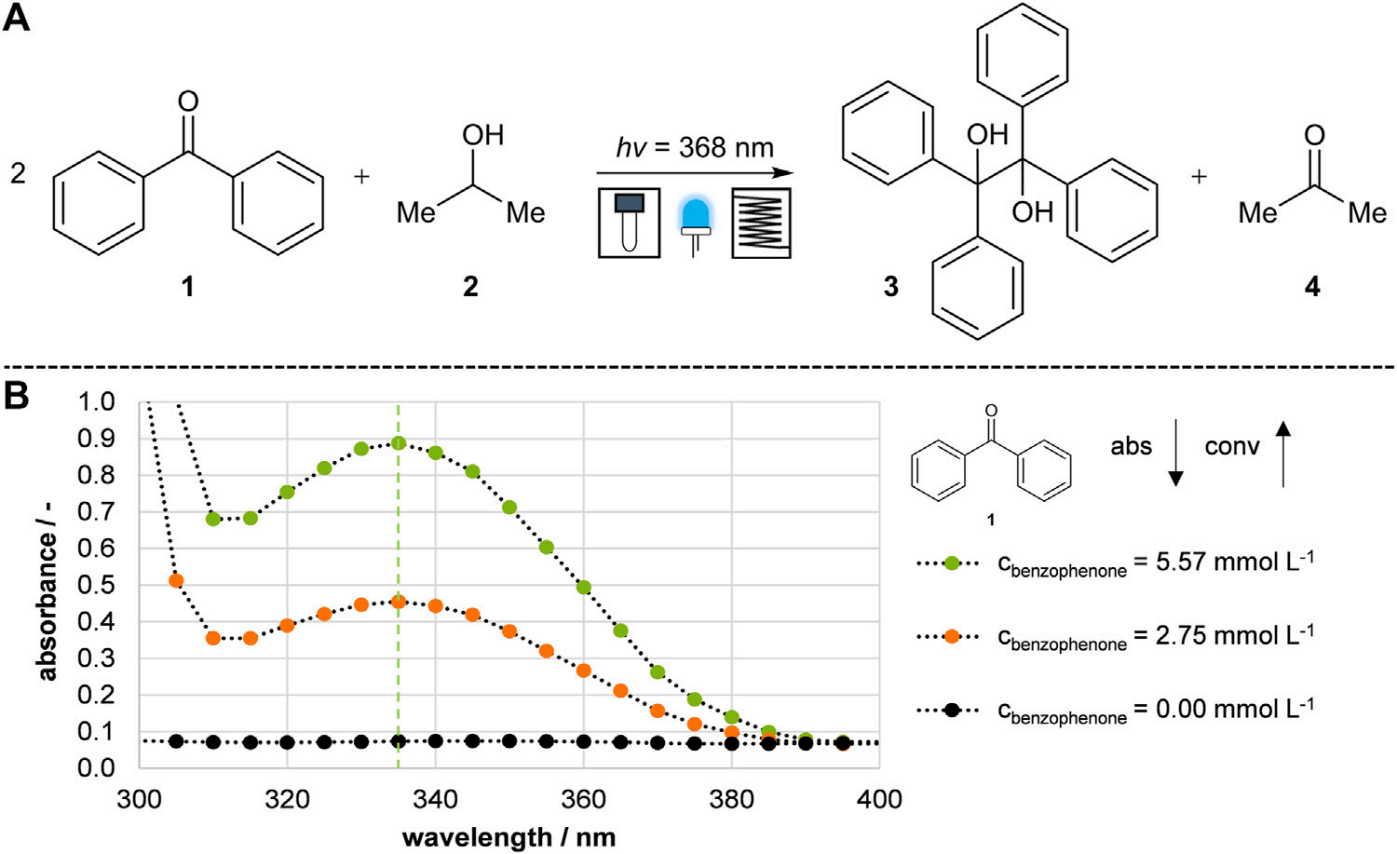
Model reaction and analysis. **(A)** Photochemical pinacol formation reaction of benzophenone to benzopinacol (Lu et al., 2001; Volpe and Podlesny, 2020). **(B)** Absorption spectra of benzophenone. The conversion was determined by the reduction of benzophenone at 335 nm.

The photochemical pinacol coupling reaction of benzophenone **1** to benzopinacol **3** was used as a model reaction. This particular reaction has already been tested in flow and batch reactors and is suitable for validating the newly developed photo-reactor prototype (Lu et al., 2001; Volpe and Podlesny, 2020). The reaction was a UV light-mediated photochemical reaction, as the reaction equation illustrated in Figure 2A, involving the generation of radicals.

Irradiation with UV light (368 nm) converted benzophenone **1**, dissolved in isopropanol **2** to a high-energy excited state and coupled to benzopinacol **3**. The reaction was completed by a short irradiation time of less than 5 min. Initially, it was tested if the conversion of benzophenone **1** to benzopinacol **3** could be observed using UV-spectrometry. Theoretically, benzophenone **1** has an absorption maximum at 335 nm in UV-Vis spectroscopy, while the reaction product **3** is UV quiescent. The absorption spectra at different concentrations are plotted in Figure 2B. The orange curve represents the absorption of a solution containing half the concentration of benzophenone **1**, and the black curve represents the base absorption of isopropanol **2**. Further experiments confirmed that the product 3 and acetone **4** do not affect the UV-Vis spectrum at 335 nm by checking the absorbance of each substance. Thus, it was possible to determine the conversion of benzophenone **1** by the decrease in UV absorbance and to compare the subsequently developed reactor prototypes.

### 3.3 Validation of prototypes

**FIGURE 3:**
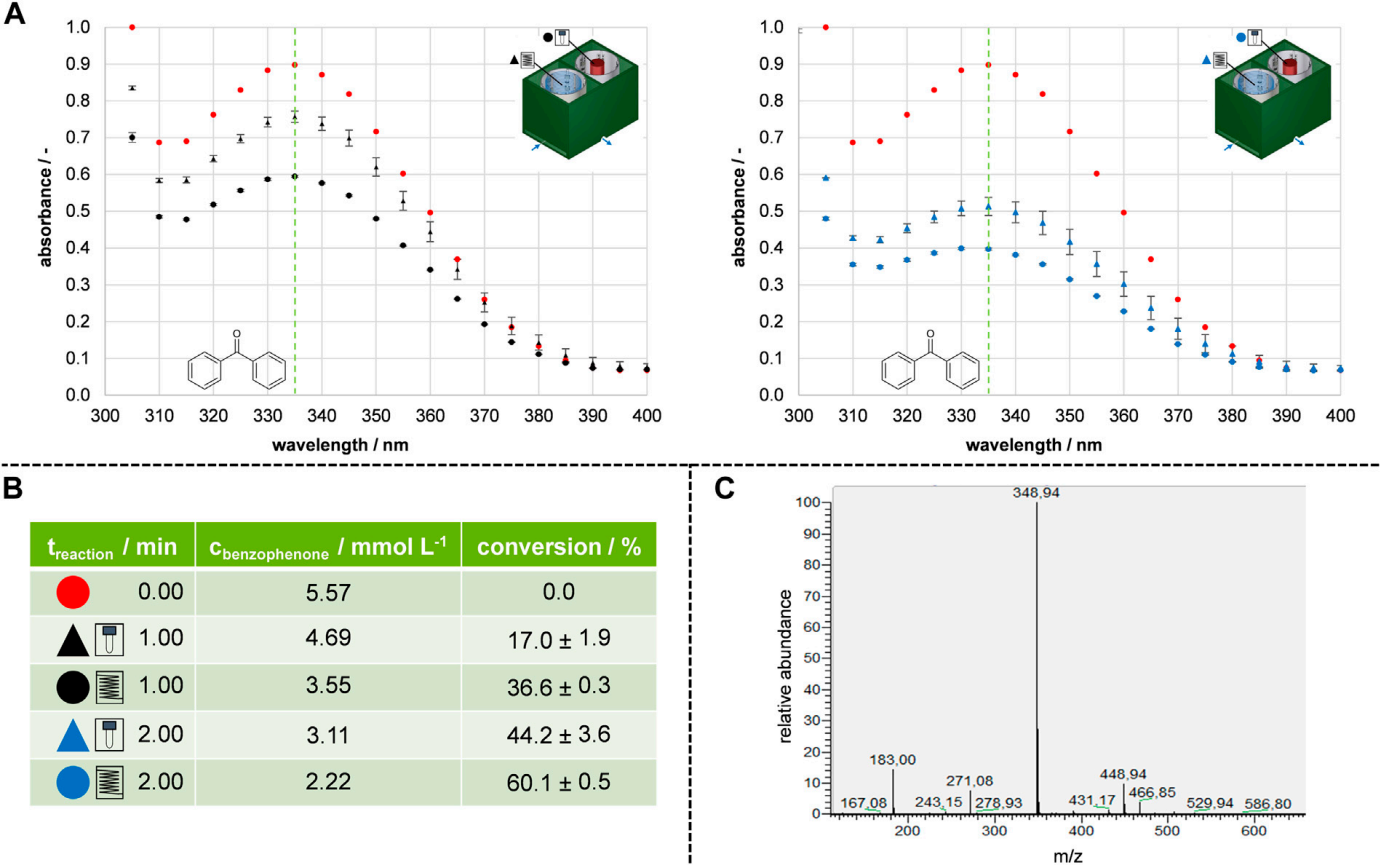
Results and validation of transferring batch to flow prototypes. **(A)** Absorption spectra of the conducted model reaction. Red represented the absorption at the initial concentration, black represented 1 min reaction time, and blue represented 2 min reaction time. **(B)** Calculated conversions and comparison of batch and flow for 1 min and 2 min reaction time. **(C)** Exclusion of side-product formation using MALDI-MS.

The model reaction from Section 3.2 was conducted to compare the prototypes, confirm successful transfer from batch to flow photochemistry and investigate reproducibility. This allowed further analysis and development of a flow photoreactor module based on the prototypes. The results of the benzopinacol formation reaction are shown in Figure 3.

Before the model reaction, the concentration of benzophenone in a stock solution was 5.57 mmol L^−1^, as indicated by the red dots on the spectrogram. After 1 min of reaction time, the flow prototype exhibited a conversion of 36.6%, which was twice as much as the batch prototype resulting in 17.0% conversion (represented by black dots in Figures 3A, B). The subsequent set of experiments showed that increasing the reaction time to 2 min further increased the conversion (represented by blue dots in Figures 3A, B). For the batch reference reactor prototype, doubling the irradiation time increased the conversion from 17.0% to 44.2%. The conversion was increased from 36.6% to 60.1% for the flow reactor prototype. The reproducible results in the flow prototype suggest that the reaction solution was uniformly irradiated, as indicated by small standard deviations. To confirm the consumption of benzophenone **1** an HPLC-MS measurement was performed (Figure 3C). Indeed, a newly formed mass (348.94 m z^−1^) was observed. In addition to the mass of the reactant (182.97 m z^−1^). This could correspond to the product of the pinacol coupling plus a subsequent pinacol rearrangement, where H_2_O was eliminated (Song et al., 2011). The study shows that both prototypes were suitable for consuming benzophenone **1**. Moreover, the flow prototype could be used to enhance absolute conversion. Therefore, a flow photoreactor module was designed based on the results of the prototype study.

### 3.4 Optimization of design parameter using the flow photoreactor module

**FIGURE 4:**
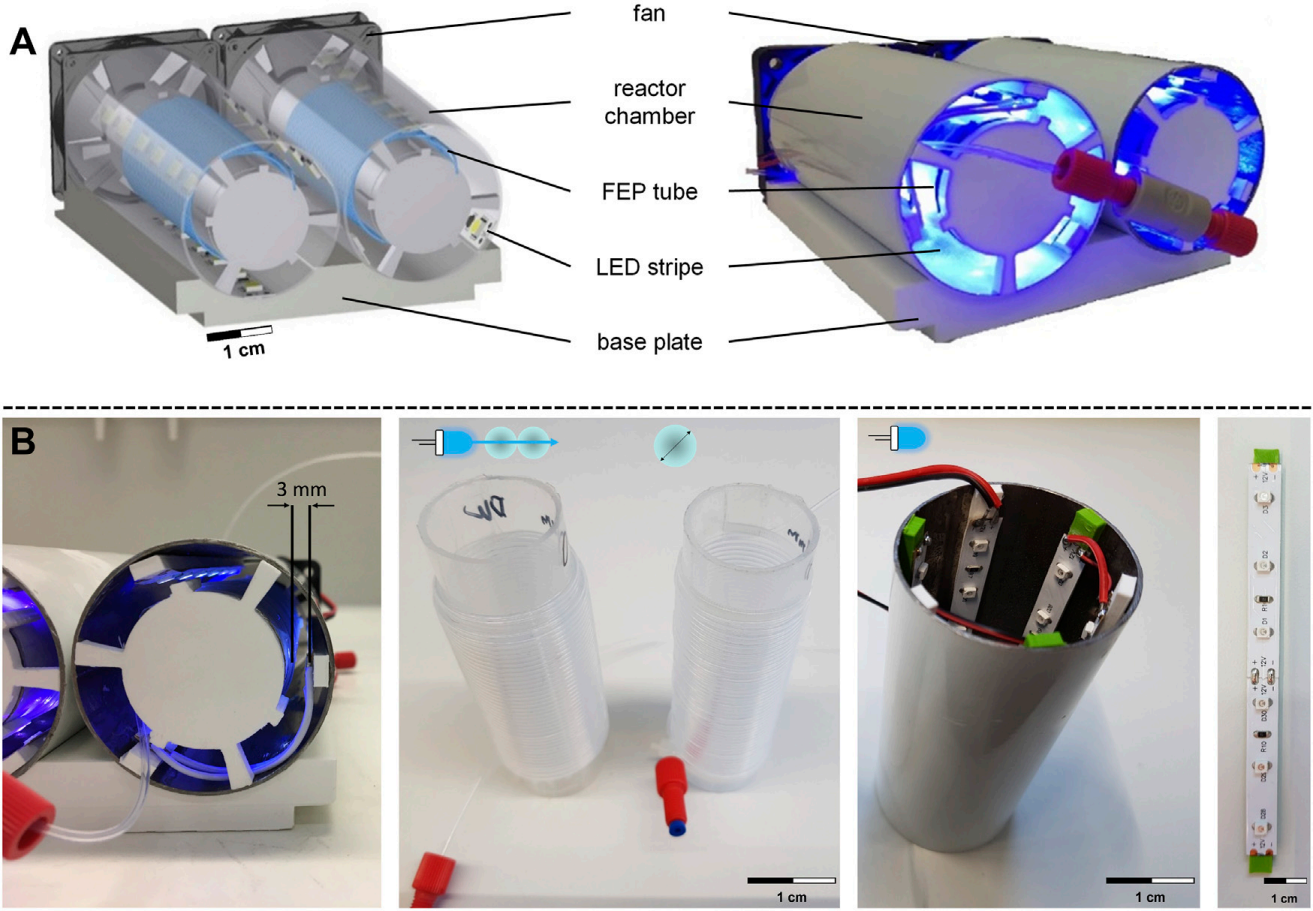
Flow photoreactor module design. **(A)** CAD model and experimental setup of the flow photoreactor module, including fan, reactor chamber, FEP tube, LED stripe, and base plate. **(B)** Structure of the irradiation chamber and spacer for LEDs on the outer tube and holder of the inner tube. Design parameters include double coiling of the FEP tube, variation of the inner diameter, and increased number of LEDs.

After successfully validating the 3D-printed prototype, a photoreactor module with a modular design was developed, as shown in Figure 4. The modular and accessible design allowed the reactor platform to be rapidly and efficiently modified to perform a wide range of photoreactions, following the methodology presented in (Bobers et al., 2020b; Bobers et al., 2020a). In contrast to the model reaction (Section 3.2) for validation (Section 3.3), the photoredox reactions envisioned for DNA-tagged reactants required visible light. Using the modular design and rapid replacement approach, the irradiation source was easily replaceable, and the reactor volume was increased by interconnecting two reactor chambers.

The base plate area of the photoreactor module was based on the outer dimensions of a 96-well microtiter plate, which could allow for automation using an automated dosage system (ADoS) established by Bobers et al. (2020b). The modular design consists of the base plate containing two cylindrical reactor chambers (Figure 4B) with an inner diameter of 50 mm. A second smaller transparent cylinder with an inner diameter of 30 mm (Figure 4B) was positioned centrally in the reactor chamber, around which a FEP capillary reactor (*L* = 4 m) was coiled. The LED stripes were inserted on the inner lateral surface of the reactor chamber using spacers (Figure 4B). The distance between the LEDs and the FEP tube was 3 mm. The spacers allowed sufficient space for convective cooling by fans and maintained a constant temperature of 26°C ± 0.5 °C in the reactor throughout the irradiation period. Additionally, a spectrometer confirmed that the wavelength range of the low-budget LEDs was 368.4 ± 0.3 nm. The modular design allowed for the rapid replacement of equipment and subsequent investigation of the following design parameters affecting the conversion of the model reaction. First, the double coiling of the FEP tube around a transparent cylinder was examined, in which the FEP tube was coiled twice around the inner transparent cylinder (Figure 4B). Second, the inner diameter of the FEP tube was altered between *d*_i_ = 0.8 mm (*V*_R_ = 2.1 mL), *d*_i_ = 0.5 mm (*V*_R_ = 0.8 mL), and *d*_i_ = 0.25 mm (*V*_R_ = 0.2 mL), while maintaining a constant outer diameter of 1.6 mm, single coiling and the same length of 4 m. Finally, the number of LEDs was doubled from 18 to 36 (Figure 4B).

**FIGURE 5:**
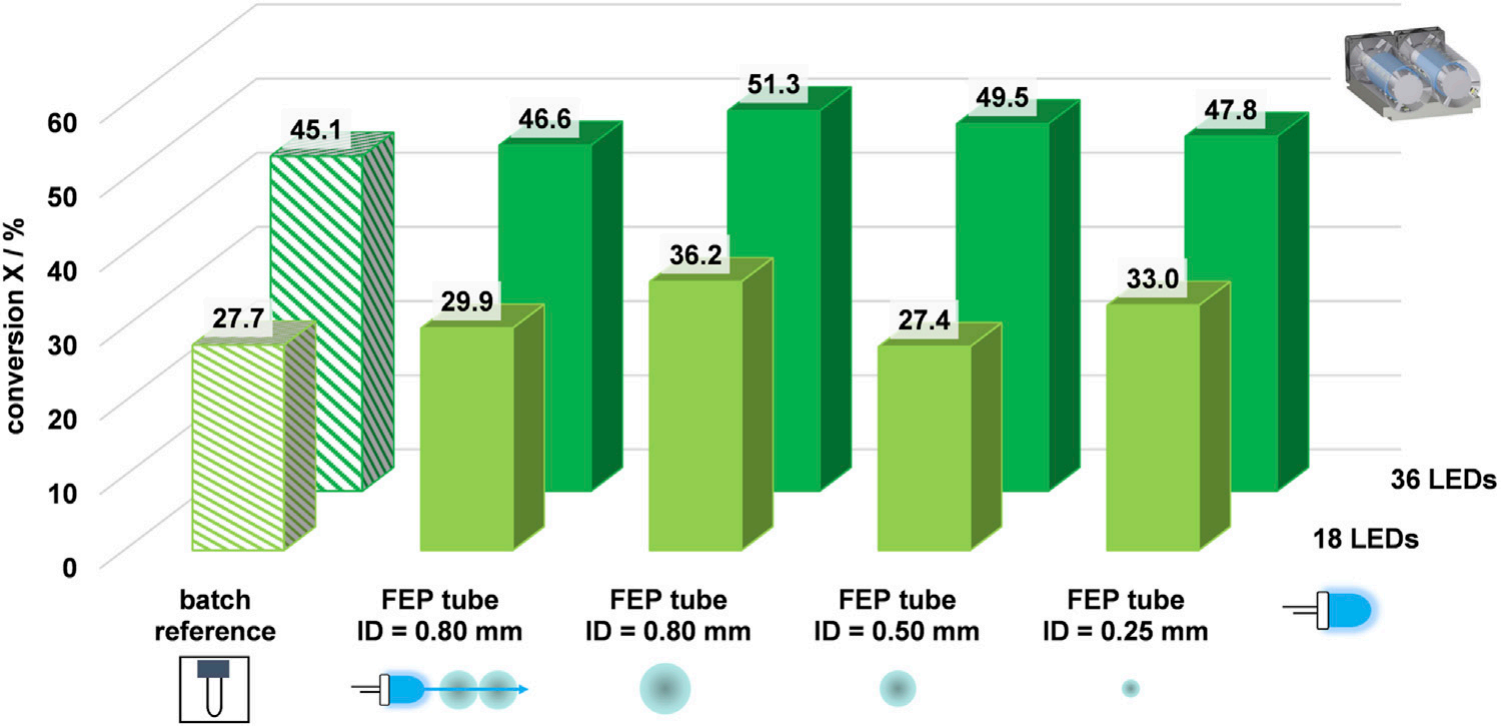
Optimization of the design parameters for enhanced conversions using the flow photoreactor module: *c*_benzophenone_ = 5.57 mmol L^−1^, *t*_r_ = 1 min, ${\dot{V}}_{ID=0.80\;mm}=1.95\;{mL\;\min}^{-1}$ , ${\dot{V}}_{ID=0.50\;mm}=0.78\;{mL\;\min}^{-1}$ , ${\dot{V}}_{ID=0.25\;mm}=0.20\;{mL\;\min}^{-1}$ .

Next, the UV light mediated photochemical reaction of benzophenone with isopropanol was conducted in the following set of experiments. The volumetric flow rate was adjusted depending on the installed capillary reactors, which vary with the inner diameter, in order to investigate the influence of these design parameters on the conversion of the model reaction for 1 min reaction time, as shown in Figure 5.

The bar plots for 18 LEDs include the batch reference with 27.7% conversion. When analyzing the inner diameters of 0.8 mm for single and double coiling, it was observed that double coiling the tube resulted in fewer photons reaching the reaction medium. Specifically, the conversion was 29.9%, and no significant increase in conversion was observed compared to batch. However, the single coiling of the largest inner diameter of 0.8 mm produced the highest conversion of 36.2% for 1 min irradiation with 18 LEDs. Varying the inner diameter did not significantly increase conversion. Furthermore, decreasing the inner diameter to 0.25 mm increases the wall thickness while maintaining the same outer diameter of 1.6 mm. In the next set of experiments, the number of LEDs used was doubled from 18 to 36. This change resulted in a significant increase in the number of photons introduced into the reaction system. As a result, all conversions of the pinacol coupling reaction were higher than with the original 18 LEDs. By doubling the number of LEDs, it was possible to increase the conversion for the batch reference from 27.7% to 45.1%. The trend was also confirmed that the flow conversion for a single coiled tube with an inner diameter of 0.8 mm was higher than the batch reference. Doubling the number of LEDs increased the flow conversion in the same way by 36%, from 36.2% to 51.3%. Additionally, it was observed that double coiling did not provide any advantages for the conversions. In fact, the conversion rate decreased slightly, as expected for model reactions conducted with smaller inner diameters. However, this demonstrated that comparable conversions were possible for smaller reactor volumes, which DELT benefits from, as reactions for DNA-encoded chemistry were typically conducted on a µL-scale in batches. In summary, the most notable impact was observed, when the number of LEDs was doubled. Conversely, varying the inner diameters had no significant impact on the conversion rate of the model reaction.

### 3.5 Recovery of DNA-tagged substrates from the flow photoreactor module

**FIGURE 6:**
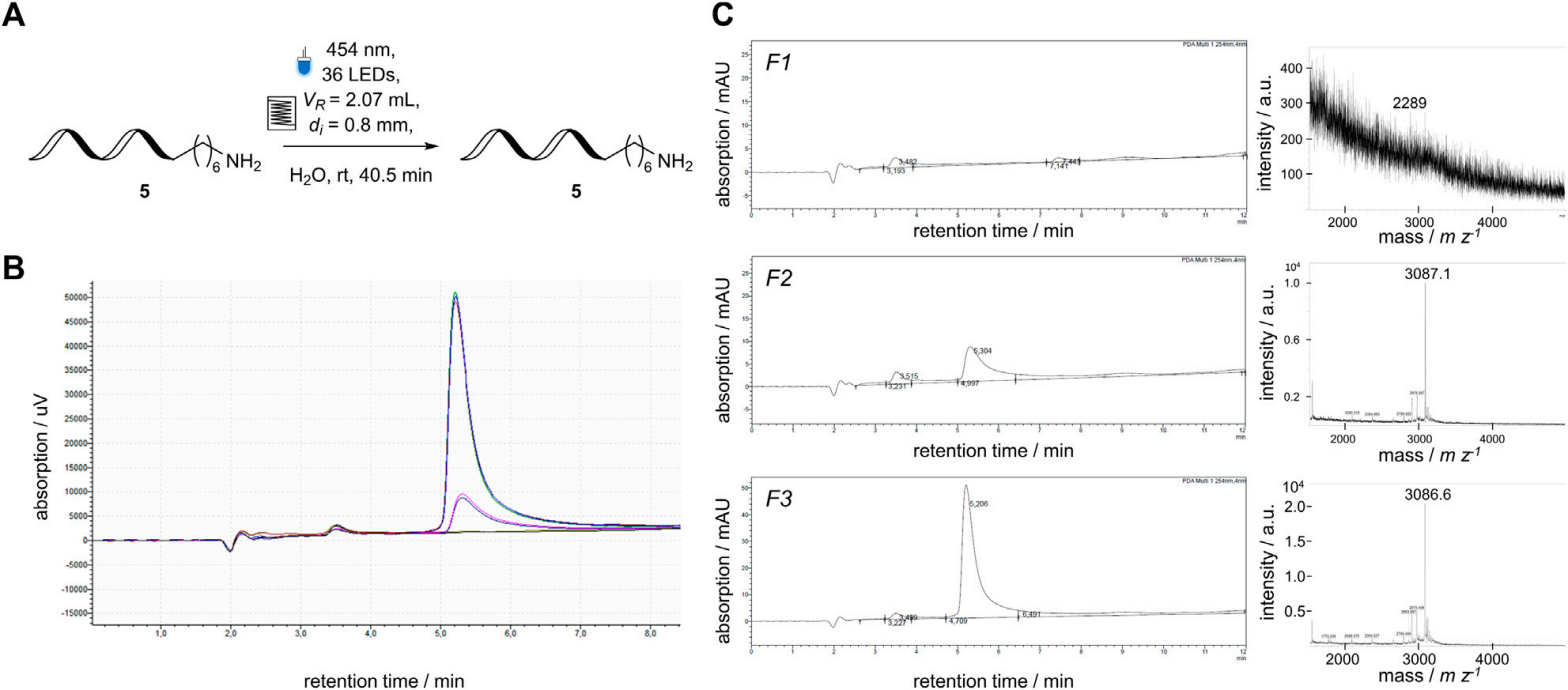
Application of a DNA-sequence (3′-TTC CTC TCC T-C6-NH_2_-5′) to the newly developed flow photoreactor module. **(A)** Reaction Scheme of single-stranded 5′-aminomodified DNA sequence 5 in 5 μmol L^−1^ concentration. **(B)** overlaid HPLC analysis of all fractions collected from the flow reactor containing the ssDNA-5 (yellow curve F1, dark blue curve F2, green curve F3, red curve F4, bright blue curve F5, pink curve F6, black curve F7), **(C)** Individual HPLC chromatograms and mass spectrum of representative fractions the DNA-sequence 5 after passing through the flow reactor, calculated mass: 3,084 m z^−1^, observed mass: 3,087 m z^−1^.

Combining flow photochemistry and DELT could be an intriguing alliance to facilitate a new platform for performing reactions on DNA. In order to see if this is accomplishable, the tailored flow photoreactor module was tested to determine whether it applies to DNA-encoded photochemistry using visible light as an irradiation source. A short single-stranded DNA-**5** (5 μmol L^−1^) was pumped through the capillary reactor, and seven identical fractions were taken manually (Figure 6A). The recovery of the ssDNA-**5** was measured by HPLC and MALDI-MS (Figures 6B, C). The DNA was recovered in five out of seven fractions without detectable damage to the DNA-**5** by the applied visible blue light with a wavelength of 454 nm (Figures 6B). However, a lower concentration of the ssDNA was detected in fractions F2 and F6 than in fractions F3 to F5 (Figures 6C). This behavior was comparable to a conventional chemical reaction conducted in a flow reaction system, as it requires a stationary operation (Plutschack et al., 2017). After the successful recovery and detection of DNA-**5**, the possibility of implementing a DELT-like reaction setup, in which reactions are conducted on a microscale (µL-scale), was planned.

**FIGURE 7:**
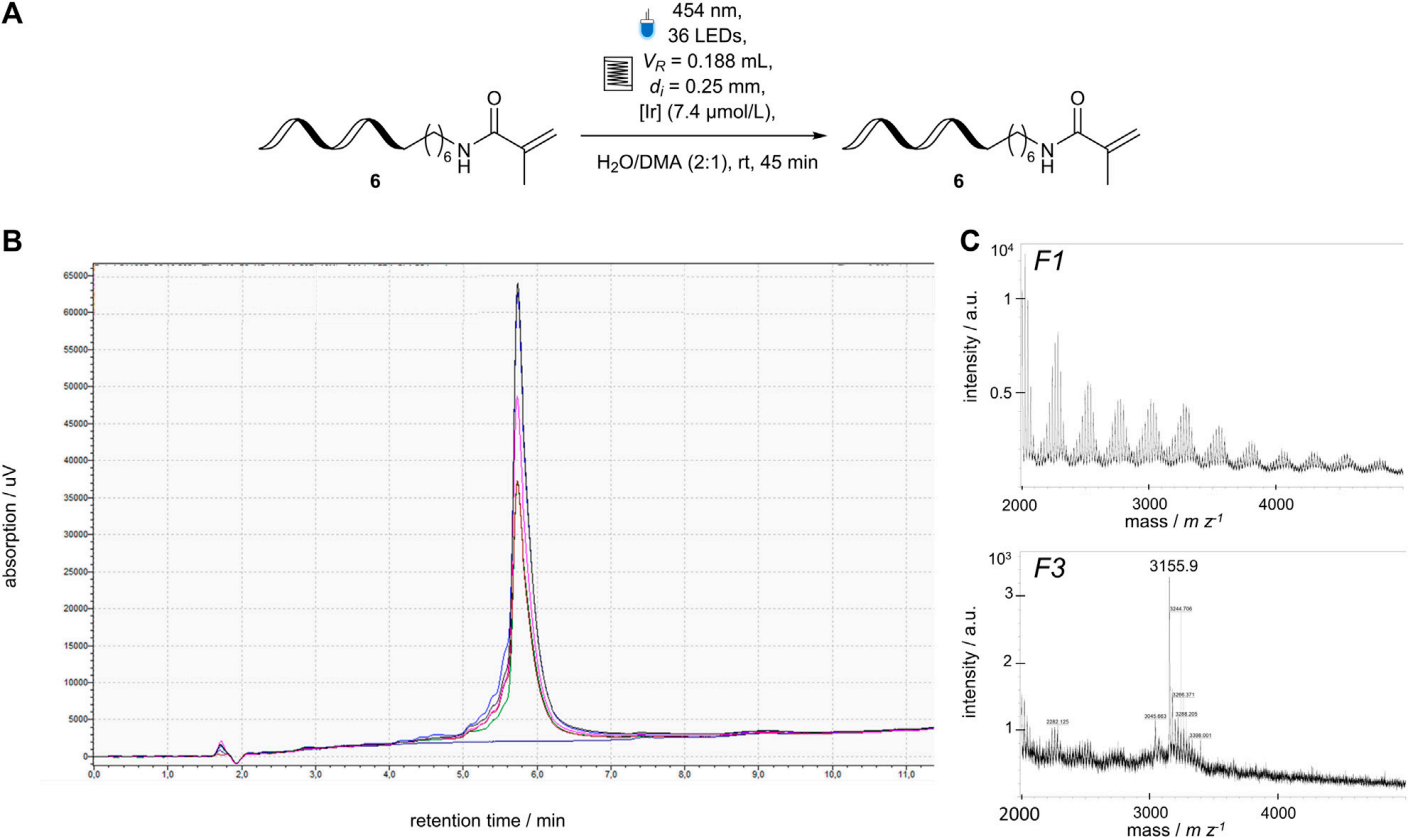
Application of a DNA-sequence 6 (3′-TTC CTC TCC T-C6-NHR-5′) to the revised flow photoreactor module, **(A)** Reaction Scheme of single-stranded 5′-aminomodified DNA sequence 6 in 1.67 μmol L^−1^ concentration [Ir]: [Ir{dFCF_3_ppy}_2_ (bpy)]PF_6_, **(B)** overlaid HPLC analysis of all fractions collected from the flow reactor containing the ssDNA-6 (dark blue curve F1, green curve F2, red curve F3, bright blue curve F4, pink curve F5, black curve F6), **(C)** mass spectrum of representative fractions the DNA-sequence 6 after passing through the flow reactor and ethanol precipitation to remove the Ir-catalyst, calculated mass: 3,152 m z^−1^, observed mass: 3,155 m z^−1^.

In order to simplify the transition of DEL-batch reactions to the flow reactor, it was concluded that the total reactor volume (*V*_R_ = 2.1 mL) was too large to facilitate a DNA-encoded reaction. It would require excessive amounts of reactants, such as DNA-tagged molecules or potential photochemical reaction mediators. Using the modular design approach of the flow photoreactor module, the inner diameter of FEP tube was decreased (*d*_i_ = 0.25 mm) to reduce the reactor volume (*V*_R_ = 0.2 mL). The capillary reactor was revalidated (Section 3.4; Figure 5) and used in a chemical reaction using DNA-tagged Michael acceptor 6 (*c* (DNA) = 1.67 μmol L^−1^) and an Ir-mediator (*c* (Ir) = 7.4 μmol L^−^) (Figure 7). After collecting and analyzing the fractions using HPLC, and MALDI-MS, the DNA-**6** was fully recovered. DNA-damage, such as DNA strand break or loss of nucleobases can be detected either by HPLC through peaks with shorter retention times or MALDI-MS by masses corresponding to cleavage products. The DNA-containing fractions did not contain significant signals corresponding to any of the described side-product (Figures 7B, C) (Potowski et al., 2019). The deviations between the manually collected fractions was slightly higher due to the smaller volume (0.2 µL) compared to the previous setup.

In summary, the previously developed flow photoreactor module was successfully applied to a DELT reaction setup. The flow photoreactor module was optimized toward a facile transfer of DNA-tagged batch reactions. The DNA-tagged substrates were fully recovered after irradiation with visible light (454 nm) and applying a photo catalyst. In further studies, it has to be shown that a photoredox reaction with DNA-tagged substrates can be performed (Kölmel et al., 2018) in the flow photoreactor module.

## 4 Conclusion

In this pivotal contribution, the performance of tailored discontinuous and continuous photo reactor prototypes was validated by conducting the benzopinacol coupling as a model reaction. The rapid prototyping study was utilized to ensure comparable and reproducible results. Subsequently, the flow prototype was transferred to a flow photoreactor module to optimize the design parameters affecting the conversion of benzopinacol. Rapid prototyping facilitated the flexible adaptation of the photoreactor module to reaction parameters for a DELT reaction system and the variety of applications with DNA-labeled substrates by rapidly replacing the required LED wavelength. Altogether, a photochemical DEL reaction was performed in the photoreactor developed by rapid prototyping and adapted to the flow reactor setup. These promising results pave the way to a wider application and scalability for continuous flow systems and fast access to a new chemical reaction space in DELT (González-Esguevillas et al., 2021; Chines et al., 2022). The photoredox reactions exhibit enormous potential for synthesizing DEL. Using photochemical flow synthesis with DNA-labeled substrates is a planned course of action. Additionally, the photoreactor module offers further research potential for the liquid-liquid two-phase flow that generates the slug flow (Dinter et al., 2023), as well as for characterizing the flow dynamics and increasing the level of automation using the automated reagent-dispensing system (Bobers et al., 2020b). Integrating the microfluidic photochemical automated flow reactor module can be beneficial for an efficient and automated DEL synthesis platform (Dinter et al., 2023).

## Data Availability

The original contributions presented in the study are included in the article/Supplementary Material, further inquiries can be directed to the corresponding authors.

## Abbreviations

ABS, acrylonitrile butadiene styrene; ADoS, automated dosage system; CAD, computer-aided design; DELT, DNA-encoded library technology; FEP, fluorinated ethylene propylene; FDM, fused deposition modeling; HPA, hydroxyl picolinic acid; HPLC, high-pressure liquid chromatography; LED, light emitting diode; MALDI-MS, matrix-assisted laser desorption/ionization mass spectrometry; PLA, polylactide.
